# Attitudes towards death in adolescents hospitalized with depressive disorder

**DOI:** 10.1192/j.eurpsy.2022.1433

**Published:** 2022-09-01

**Authors:** M. Bebchuk, D. Dovbysh, S. Timoshenko, R. Rabadanova, Y. Zhorina, A. Diachenko, M. Talmach, G. Fakhretdinova

**Affiliations:** 1 Scientific-Practical Children’s and Adolescents Mental Health Center n.a. G. Sukhareva, Moscow Department of Health Care, Clinical Psychology, Moscow, Russian Federation; 2 Federal State Autonomous Educational Institution of Higher Education I.M. Sechenov First Moscow State Medical University under the Ministry of Health of the Russian Federation (Sechenov University), Pedagogy And Clinical Psychology, Moscow, Russian Federation

**Keywords:** Adolescents, attitudes towards death, depressive disorder

## Abstract

**Introduction:**

The study of attitudes towards death in patients of different nosological groups is an urgent task for modern science. It becomes especially relevant when working with adolescents with severe depressive disorder: for many of them, thoughts about death in various forms become the main reason for contacting specialists and the most subjectively painful symptom.

**Objectives:**

Revealing the characteristics of attitudes towards death in adolescents with severe depressive disorder.

**Methods:**

The study involved 135 adolescents (12-17 years old) with depressive disorder, hospitalized in a psychiatric hospital. Participants completed the following methods: Hamilton Rating Scale for Depression, Columbia Suicide Severity Rating Scale, Death Attitude Profile-Revised, Fear of Personal Death Scale, Death Anxiety Scale.

**Results:**

The severity of depressive symptoms is significantly associated with the “death-as-flight” scale (r = 0.639, p = 0.000). The values on the “fear of death” scale are positively associated with the indicators on the scales “death anxiety” (r = 0.432 p = 0.025), “consequences of death for the individual” (r = 0.658, p = 0.000), “transcendental consequences of death” (r = 0.711, p = 0.000), “the consequences of my death for loved ones” (r = 0.496, p = 0.008). Indicators on the “active death search” scale are negatively associated with indicators on the “neutral acceptance of death” scale (r = -0.503, p = 0.007) and positively with the “fear of oblivion” scale (r = 0.432, p = 0.024).

**Conclusions:**

The attitude towards death in adolescents with depressive disorder has a pronounced specificity, which can become one of the targets of psychotherapeutic work.

**Disclosure:**

No significant relationships.

